# Periodic Exposure of Plasma-Activated Medium Alters Fibroblast Cellular Homoeostasis

**DOI:** 10.3390/ijms23063120

**Published:** 2022-03-14

**Authors:** Pradeep Bhartiya, Neha Kaushik, Linh N. Nguyen, Sander Bekeschus, Kai Masur, Klaus-Dieter Weltmann, Nagendra Kumar Kaushik, Eun Ha Choi

**Affiliations:** 1 Department of Electrical and Biological Physics, Plasma Bioscience Research Center, Kwangwoon University, Seoul 01897, Korea; pradeepindian65@gmail.com (P.B.); nhatlinhusth@gmail.com (L.N.N.); 2 Department of Biotechnology, College of Engineering, The University of Suwon, Hwaseong 18323, Korea; neha.bioplasma@gmail.com; 3 Laboratory of Plasma Technology, Institute of Materials Science, Vietnam Academy of Science and Technology, 18 Hoang Quoc Viet, Hanoi 100000, Vietnam; 4 ZIK Plasmatis, Leibniz Institute for Plasma Science and Technology, 17489 Greifswald, Germany; sander.bekeschus@inp-greifswald.de (S.B.); kai.masur@inp-greifswald.de (K.M.); weltmann@inp-greifswald.de (K.-D.W.)

**Keywords:** cellular stability, cold atmospheric plasma, DNA damage response, dermal fibroblasts

## Abstract

Excess amounts of redox stress and failure to regulate homeostatic levels of reactive species are associated with several skin pathophysiologic conditions. Nonmalignant cells are assumed to cope better with higher reactive oxygen and nitrogen species (RONS) levels. However, the effect of periodic stress on this balance has not been investigated in fibroblasts in the field of plasma medicine. In this study, we aimed to investigate intrinsic changes with respect to cellular proliferation, cell cycle, and ability to neutralize the redox stress inside fibroblast cells following periodic redox stress in vitro. Soft jet plasma with air as feeding gas was used to generate plasma-activated medium (PAM) for inducing redox stress conditions. We assessed cellular viability, energetics, and cell cycle machinery under oxidative stress conditions at weeks 3, 6, 9, and 12. Fibroblasts retained their usual physiological properties until 6 weeks. Fibroblasts failed to overcome the redox stress induced by periodic PAM exposure after 6 weeks, indicating its threshold potential. Periodic stress above the threshold level led to alterations in fibroblast cellular processes. These include consistent increases in apoptosis, while RONS accumulation and cell cycle arrest were observed at the final stages. Currently, the use of NTP in clinical settings is limited due to a lack of knowledge about fibroblasts’ behavior in wound healing, scar formation, and other fibrotic disorders. Understanding fibroblasts’ physiology could help to utilize nonthermal plasma in redox-related skin diseases. Furthermore, these results provide new information about the threshold capacity of fibroblasts and an insight into the adaptation mechanism against periodic oxidative stress conditions in fibroblasts.

## 1. Introduction

Skin cells are frequently exposed to exogenous stress and achieve homeostasis through not entirely known mechanisms [1,2]. Generally, skin maintenance depends on the continual proliferation and differentiation of skin cells corresponding to the skin renewal potential. This potential slows as tissue becomes older or is exposed to stress resulting from reactive oxygen and nitrogen species (RONS), telomere shortening, mitochondrial DNA mutations, as well as hormonal changes [3,4]. Skin renewal also depends on the interaction between epidermal and dermal cells. The epidermis comprises mainly keratinocytes and other cells such as melanocytes and Merkel and Langerhans cells [5,6,7,8]. The dermis is structurally more complex and consists of fibroblasts and their extracellular matrix, sweat glands, vasculature, nerves, and lymphatic vessels [9,10]. Fibroblasts perform key functions in wound healing, contribute to cutaneous infection control, and communicate with neighboring cells [11]. The papillary dermis is considered essential for epidermal organization and faces a more substantial impact of redox changes and intrinsic skin aging, eventually affecting skin renewal functionality [12,13]. Molecular characterization and detailed knowledge of fibroblast cell functions and interactions under redox stress are integral for understanding skin homeostasis in aging and various skin diseases.

Nonthermal atmospheric plasma (NTP) devices have recently been used in dermatology owing to various medical benefits [14]. Among them, the most promising and efficient action is observed as an antibacterial effect in skin diseases and the acceleration of wound healing processes. Still, the mechanism underlying NTP-mediated action on skin tissue is not fully understood. There is growing consensus on the significant role of RONS in plasma-induced effects [15,16,17,18,19,20]. Elevated RONS levels act as double-edged swords involved with cellular regulatory functions ranging from stimulating cell proliferation to inhibition by inducing cell death [21]. RONS levels have been reported to induce DNA damage, interact with DNA, and modulate the DNA damage responses [22,23]. Cells with increased RONS levels were reported to undergo cell cycle arrest in S and G2/M phases [24]. To protect against harmful effects and maintain the RONS balance physiologically, cells utilize an intracellular defense system to eliminate excess endogenous and exogenous reactive species [4,21,25]. The impact of impaired RONS balance due to acute oxidative stress has been reported in skin cells [26,27]. The cellular and molecular changes in epidermal cells (keratinocytes) following repeated exposure over three months to a cold plasma-treated cell culture medium were thoroughly investigated before [28]. The study provided insights on redox regulation and generated an opportunity to investigate skin functions in the redox stress model, which was recently translated into several animal models of wounded and unwounded skin [29,30,31]. However, the effect of mild oxidative stress has not been thoroughly investigated, especially in fibroblasts.

In this study, we investigated the chronic effects of NTP in fibroblast cells using the redox model described previously [28]. Plasma-activated medium (PAM) was used periodically to induce mild redox stress in GM00637 (GM) human fibroblast cells and assess key aspects of stress regulation in chronic oxidative stress conditions. We hypothesized that periodic stress would induce several changes that are different from acute stress conditions. Understanding these changes in fibroblasts would emphasize the distinct machinery employed by cells when exposed to oxidative stress for long periods of NTP therapy intervals. Here, we focused on the changes occurring in apoptotic processes, cellular energetics, cell cycle machinery, and cellular antioxidant defense responses. Knowledge of such changes provides essential information for oxidative stress-induced skin diseases and their therapy using NTP.

## 2. Results

### 2.1. Physical Characteristics of Soft Jet Plasma

**Figure 1:**
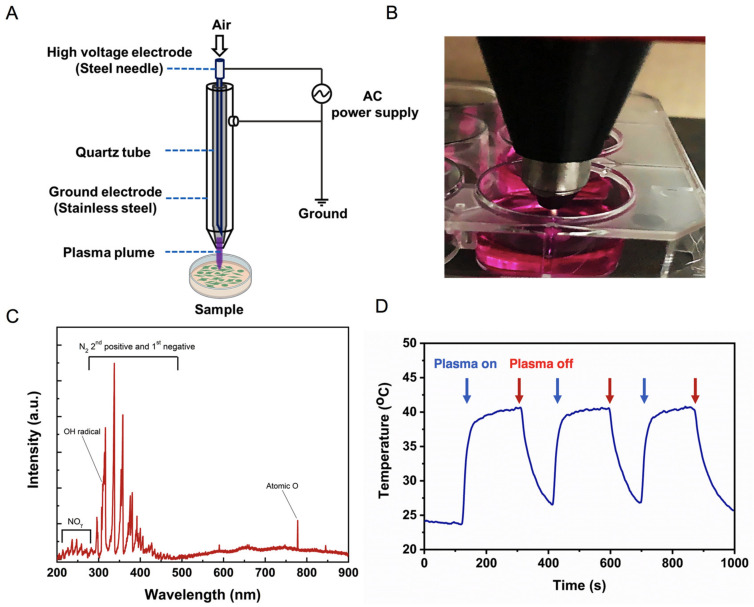
Physical characteristics of the soft jet plasma device. (**A**) Schematic representation of soft jet plasma device used in this study. (**B**) Photograph of the performing soft jet plasma. (**C**) Optical emission spectrum of the plasma jet, showing emissions of NO, the N_2_ second positive system (N_2_ SPS) and the N_2_ first negative system (N_2_ FNS), OH radical, and atomic O. (**D**) Temperature profile of the plasma jet plume during operation.

Figure 1A,B demonstrate the schematic and photograph of the plasma jet system used in this study, respectively. The plasma jet was constructed by inserting a steel syringe needle inside a quartz tube, which was protected by a stainless steel cover. Natural air was supplied through the needle as the working gas and was controlled at a flow rate of 1 L per minute. The needle acted as the high-voltage electrode, while the stainless steel cover was considered the ground electrode. An AC voltage of 2.2 kV with a frequency of 42 kHz was applied between the high-voltage and ground electrodes with a fixed duty cycle of 7%. The optical emission spectrum (OES) of the plasma jet was recorded by using an HR4000-UV-NIR spectrometer (Ocean Optic, Dunedin, FL, USA) (Figure 1C). Because the plasma jet was operated in ambient air conditions, several RONS were observed in the OES. For instance, emission signals of the NO-γ band at 200–280 nm; the N_2_ second positive system/N_2_ (${\;C}^{3}\Pi_{u}\;\to {\;B}^{3}\Pi_{g}$) and the N_2_ first negative system/N_2_+ ($B^{2}\Sigma_{u}^{+}$ → $X^{2}\Sigma_{g}^{+}$) at 310–450 nm; OH radical at 309 nm; and atomic O at 777 nm were detected. These plasma-produced RONS are the main components that contribute to the complex plasma chemistry in both plasma and bulk liquid [32,33,34]. For instance, the •OH radicals in the plasma effluent are formed by the dissociation of water molecules in the ambient environment:

$$e\;+{\;H}_{2}O\;\to H\;+•\mathrm{OH}\;+\;e\;$$

Plasma-produced OH radicals recombine to form gas-phase H_2_O_2_, which can be transported into the liquid medium [35]. Moreover, solvated OH radicals can also recombine to form liquid-phase H_2_O_2_ [36]:

$$•{\mathrm{OH}}_{\left(g\right)}+•{\mathrm{OH}}_{\left(g\right)}\to H_{2}O_{2}{}_{\left(g\right)}\to H_{2}O_{2}{}_{\left(\mathrm{aq}\right)}$$

$$•{\mathrm{OH}}_{\left(\mathrm{aq}\right)}+•{\mathrm{OH}}_{\left(\mathrm{aq}\right)}\to H_{2}O_{2}{}_{\left(\mathrm{aq}\right)}$$

Nitric oxide (NO) is a nitrogen reactive species that is produced by the collision of plasma-produced RONS with each other or with molecular oxygen and molecular nitrogen in ambient air [37]:

$$•O\;+{\;N}_{2}^{*}\to \;\mathrm{NO}\;+•N$$

$$O_{2}+•N\to \;\mathrm{NO}\;+•O$$

•OH +•N → NO+•H

•O +•N + M → NO+ M

where M is the third body molecule. In the gas phase, NO can be further oxidized to NO_2_^−^ or nitrate NO_3_^−^, which can then be solvated into the bulk liquid [37]:

$${\mathrm{NO}}_{\left(g\right)}+\;\mathrm{OH}\;\to H^{•}+{\;\mathrm{NO}}_{2\left(g\right)}^{-}\;\to {\;\mathrm{NO}}_{2\left(\mathrm{aq}\right)}^{-}$$

$${\mathrm{NO}}_{2\left(g\right)}^{-}+\mathrm{OH}\;\to H^{•}+{\;\mathrm{NO}}_{3\left(g\right)}^{-}\to {\;\mathrm{NO}}_{3\left(\mathrm{aq}\right)}^{-}$$

### 2.2. Single and Periodic Exposure to PAM Leads to Reduced Viability of Fibroblasts

**Figure 2:**
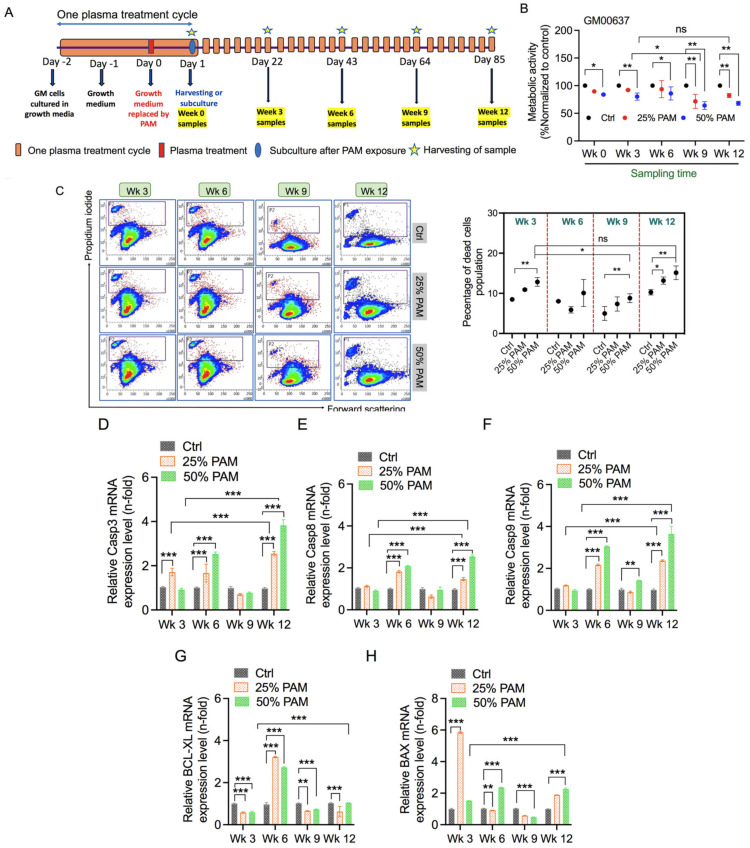
Exposure to PAM reduces the viability of GM fibroblast cells. (**A**) Schematic representation of the study and experimental plan. (**B**) Bar graph representing metabolic activity of fibroblasts after periodic PAM exposure for weeks (Wk). (**C**) Representative flow cytometry dot plots and respective bar graphs indicating terminal cell death. Gene expression profiles as measured by qPCR for (**D**) Casp3, (**E**) Casp8, (**F**) Casp9, (**G**) BCL-XL, and (**H**) BAX. Statistical significance was evaluated using Dunnett’s or Tukey’s multiple comparison test with two-way ANOVA and is indicated as * *p* < 0.05, ** *p* < 0.01, and *** *p* < 0.001.

The objective of this study was to generate mild redox stress conditions and repeat them periodically for an extended period of investigation. The experimental plan was designed for up to 12 weeks to determine changes occurring at different stages of the study span. Preliminary experiments were carried out to determine the optimal plasma exposure time and volume of PAM to be used. Figure S1 indicates that 200 s exposure time in 3 mL of medium is the least cytotoxic but has a significant effect on cellular metabolic activity and can be used for the scheduled experiment. To begin the scheduled experiment, GM cells were cultured in complete growth media for two days, as shown in Figure 2A. On the third day, the growth medium was replaced with PAM. Cells were sub-cultured after 24 h incubation on the fourth day. To establish the concentration of PAM required to induce oxidative stress, we exposed the GM cells to various concentrations of PAM (0%, 25%, 50%, and 100%) and measured metabolic activity using alamarBlue assays. Undiluted PAM (100%) concentration was observed to be highly cytotoxic (47% reduction in metabolic activity), as shown in Figure S2, and not suitable for the long experimental plan in this study. The 50% PAM dilution showed a mild cytotoxic effect (18% reduction), as shown in Figure 2B, and was found to be ideal for inducing oxidative stress and simultaneously allowing us to culture cells for extended periods. The reduction in metabolic activity was observed consistently after each cycle of PAM treatment compared to the respective control. This reduction was further enhanced owing to periodic redox stress until the ninth week but reverted to nonsignificant levels compared to the initial weeks. These data were further reinforced by measuring propidium iodide (PI) uptake in cells, as shown in Figure 2C. These results indicated that periodic exposure of PAM to GM cells consistently induces terminal cell death. However, at week 12 under periodic stress conditions, the terminal cell death rates were not altered significantly compared to the initial weeks. To determine if cells were taking the apoptotic path, we quantitatively measured mRNA levels of key apoptotic genes at weeks 3–12 (chronic stress) by real-time PCR. As shown in Figure 2D–H, apoptotic genes were upregulated in PAM-treated groups and remained elevated in chronic stress conditions. Our results indicate that both 25% and 50% PAM treatment could trigger biochemical processes of metabolism and apoptosis without affecting overall survival. To understand these results further for conclusive comprehension, we investigated the intermediate weeks 1.5, 4.5, 7.5, and 10.5 for cell death regulation. We observed similar trends regularly at intermediate week time points, indicated by the consistent reduction in metabolic activity of fibroblasts (Supplementary Figure S5A). Furthermore, the expression patterns of Casp8 and Casp9 were found to be similar (Supplementary Figure S5B,C). In our study, week 0 may represent acute stress, whereas the following periodic PAM treatments may induce chronic stress. Here onwards, we investigated major cellular processes and compared the difference between the onset of chronic stress (week 3) with final stages (week 12) of redox stress.

### 2.3. PAM Exposure Increases Reactive Species Accumulation and Triggers Cellular Antioxidant Proteins

**Figure 3:**
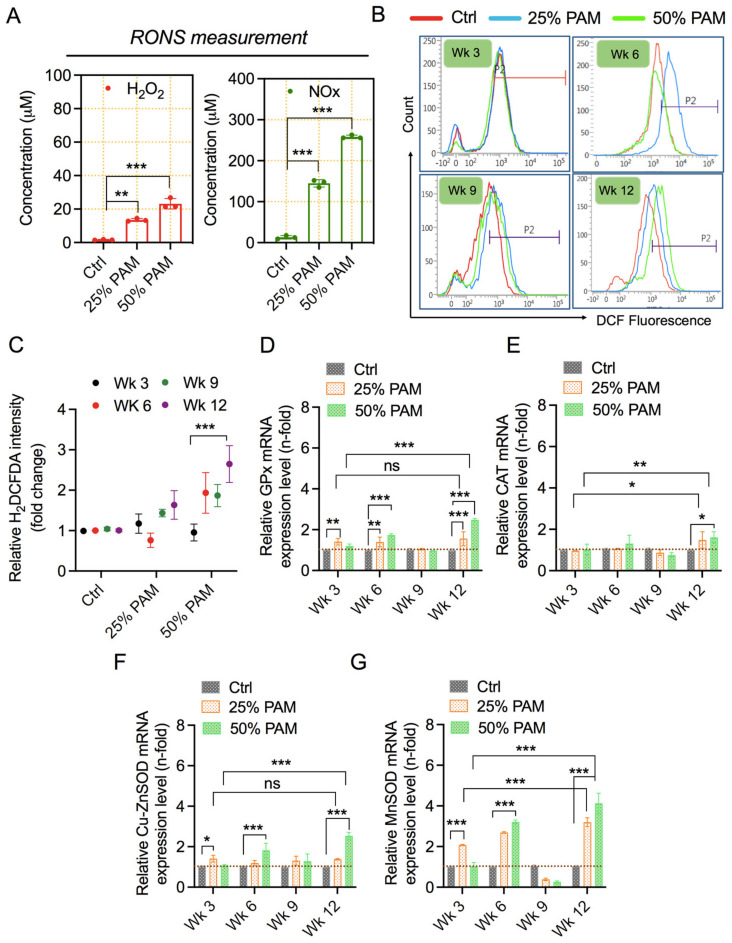
Exposure to PAM induces RONS levels and increases the cellular antioxidant system. (**A**) The relative concentration of reactive species in the medium after PAM exposure. Statistical significance was evaluated using Dunnett’s multiple comparison test with one-way ANOVA analysis. Representative spectra (**B**) and a bar graph (**C**) representing H_2_-DCF-DA levels in fibroblasts after periodic PAM exposure for weeks (Wk). Statistical significance was evaluated using Tukey’s multiple comparison test with two-way ANOVA. Gene expression profile of antioxidant enzymes (**D**) GPx, (**E**) catalase, (**F**) CuZnSOD, and (**G**) MnSOD. Statistical significance was evaluated using Dunnett’s or Tukey’s multiple comparison test with two-way ANOVA and is indicated as * *p* < 0.05, ** *p* < 0.01, and *** *p* < 0.001.

NTP is reported to generate various reactive species in the medium [38,39,40]. Firstly, we focused on H_2_O_2_ and NO_x,_ which are generated in PAM. Figure 3A and Figure S3 show the concentrations of H_2_O_2_ and NO_x_ levels measured by quantichrom commercial kits in PAM (0%, 25%, and 50%) and PAM (0% and 100%), respectively. Next, we determined the levels of intracellular RONS inside cells after periodic oxidative stress using H_2_-DCFDA staining and analysis using flow cytometry. The results shown in Figure 3B,C indicate that intracellular RONS increases in cells from the sixth week, being accumulated in subsequent weeks. RONS levels are under tight regulation by intracellular antioxidant proteins. To ascertain their role in repeated stress conditions, we measured the mRNA expression levels of key antioxidant enzymes by qPCR. Expression of major antioxidant enzymes (glutathione peroxidases-GPx, superoxide dismutase 1-CuZnSOD, and superoxide dismutase 2-MnSOD) but not catalase was enhanced by periodic exposure to PAM, as shown in Figure 3D–G. Catalase mRNAs were significantly increased at the final stages of periodic stress. Furthermore, we analyzed the samples at intermediate weeks 1.5, 4.5, 7.5, and 10.5. We evaluated the expression pattern of CuSOD and found it to be comparatively similar to other time points (Supplementary Figure S4D). This may reinforce the results obtained earlier and suggest altered regulation of antioxidants after week 9.

### 2.4. Periodic PAM Exposure Effect on Fibroblast Cell Energetics

**Figure 4:**
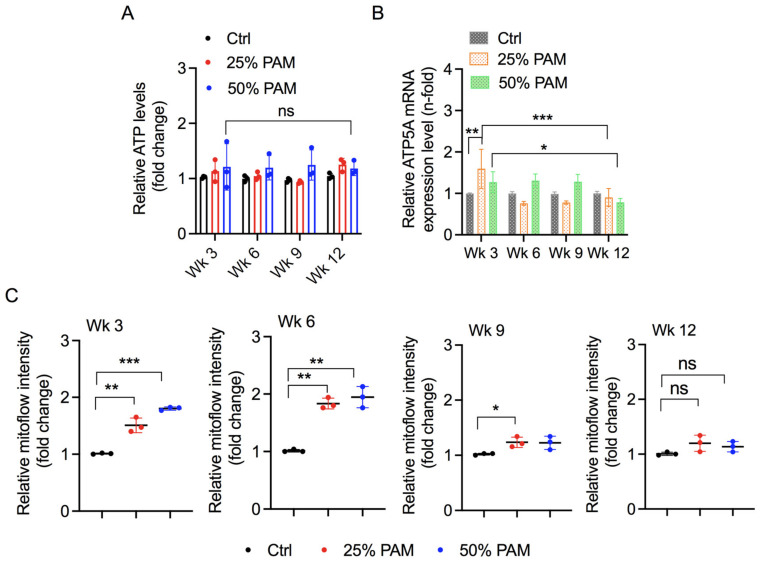
Effect of periodic PAM exposure on cellular energetics. (**A**) ATP levels in fibroblast cells were exposed periodically to PAM. Statistical significance was evaluated using Dunnett’s multiple comparison test with two-way ANOVA analysis. (**B**) mRNA levels of ATP5A gene. Statistical significance was evaluated using Dunnett’s or Tukey’s multiple comparison test with two-way ANOVA analysis. (**C**) Mitoflow intensity levels in fibroblast after periodic PAM exposure across weeks (Wk). Statistical significance was evaluated using Dunnett’s multiple comparison test with one-way ANOVA and is indicated as * *p* < 0.05, ** *p* < 0.01, and *** *p* < 0.001.

Cell proliferation and death are largely dependent on cellular metabolism and energetics within the cell [41]. To determine how cells modulate their energy needs to nullify acute and chronic oxidative stress, we measured the ATP levels at week 0 and followed their changes over the investigation period. As shown in Figure 4A and Figure S4, PAM exposure did not reduce ATP levels. ATP synthesis was not affected over the investigation period after periodic PAM exposure, as quantification of ATP5A transcript expression remained unaffected (Figure 4B). Cellular energy production and regulation are dependent on mitochondrial structural and functional integrity. To this end, we measured the mitochondrial membrane potential of cells indicative of structural integrity of mitochondria using mitoflow stain and analysis by FACS. Figure 4C shows the changes in mitochondrial membrane potential at different weeks after periodic exposure to PAM. Acute stress induces changes in mitochondrial membrane potential, and such change is consistent after periodic PAM exposure as time progresses. Mitoflow intensity was observed to be significantly high in PAM-exposed groups at week 3 and week 6. Compared to week 3 and week 6, this increase was low in week 9 and week 12 in the PAM-treated groups compared to respective untreated control groups.

### 2.5. Periodic PAM Exposure Induces the DNA Damage Response and Cell Cycle Arrest in Fibroblast Cells

**Figure 5:**
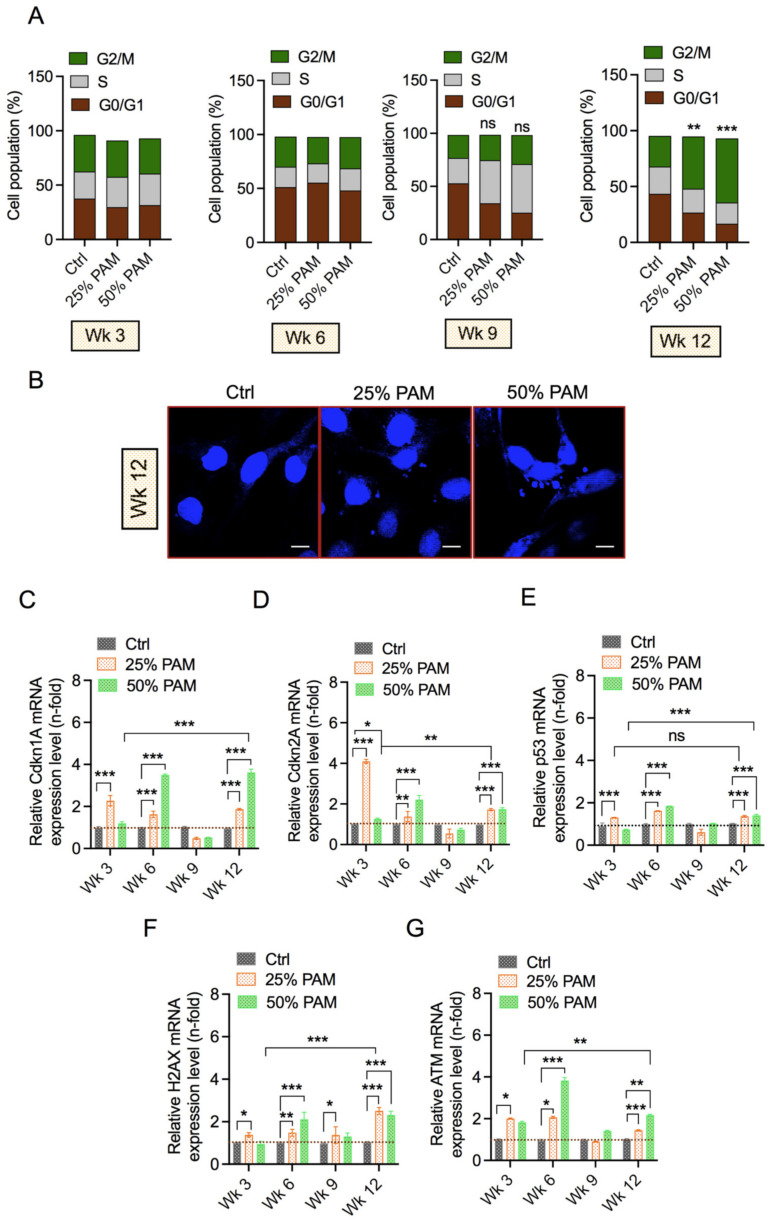
Evaluation of the DNA damage response and cell cycle phases after periodic PAM exposure in fibroblast cells. (**A**) Representative cell cycle phases in fibroblast cells after PAM exposure for weeks (Wk). Significance has been shown among various groups considering G2/M changes. Statistical significance was evaluated using Dunnett’s multiple comparison test with one-way ANOVA. (**B**) Representative confocal images of fibroblast cells stained with DAPI indicate changes in nuclear morphology. mRNA levels of cell cycle markers. (**C**) Cdkn1A, (**D**) Cdkn2A, and (**E**) p53. mRNA levels of DNA damage markers (**F**) H2AX and (**G**) ATM. Scale bar 100 µM. Statistical significance was evaluated using Dunnett’s or Tukey’s multiple comparison test with two-way ANOVA and is indicated as * *p* < 0.05, ** *p* < 0.01, and *** *p* < 0.001.

Cellular growth is largely dependent on tight regulation of the cell cycle. NTP can effectively inhibit cell proliferation by arresting cells in the S and G2/M phases, which can correlate to cytotoxicity [38,39]. DNA damage and cell cycle arrest are associated with the presence of excessive reactive species [24]. To determine the cell cycle machinery changes in periodic oxidative stress conditions, we used the PI staining method to assess cell cycle arrest over the long investigation course through flow cytometry analysis. The results from Figure 5A indicate non-significant changes in cell cycle phases in the initial weeks. However, periodic PAM exposure induced accumulation of cells in G2/M phases eventually at week 12, indicating extensive cell cycle arrest. We also observed changes in nuclear morphology due to periodic stress (Figure 5B). Although these changes in nuclear morphology are not as definitive as micronuclei, they raised curiosity about DNA damage. Cell cycle arrest in G2/M phases can be induced due to DNA damage during replication in the S phase or inefficient repair downstream of replication. To further understand this case, we assessed the expression of key cell cycle regulator proteins and marker genes indicative of the DNA damage response by qPCR. The upregulation of cell cycle regulators—Cyclin Dependent Kinase Inhibitor 1A (*cdkn1A*) and Cyclin Dependent Kinase Inhibitor 1A (*cdkn2A*)—at initial and final investigation periods indicates impaired cell cycle regulation (Figure 5C,D). The product of TP53, p53, helps regulate the cell cycle by inducing arrest or apoptosis, provided repair is not possible. As shown in Figure 5E, p53 expression is concomitantly regulated with cdkn1A and cdkn2A, indicating a role in PAM-induced cell cycle arrest. Expression of the DNA damage response (histone 2A.X, Serine/Threonine Kinase ATM) was increased significantly in PAM-exposed groups in the earlier and final weeks of the investigation (Figure 5F,G). This upregulation could be simply due to oxidative stress or the combined effect of oxidative stress with DNA damage [20]. Taken together, occurrence of the DNA damage response could be the most plausible reason for the induction of cell cycle arrest and/or impaired proliferation in our cell system.

## 3. Discussion

The selective eradication of cancer cells without damaging nonmalignant cells is one of the hypotheses in medical technology. However, NTP can play a significant role in the modulation of several responses affecting cellular homeostasis and tissue function of both types of cells. Owing to their location in the skin, nonmalignant skin cells experience stress repeatedly by intrinsic and extrinsic redox changes. Fibroblasts are key for skin architecture, and their interaction with other cell types facilitates necessary physiological functions. In this study, we investigated the effect of periodic oxidative stress on fibroblasts for three months by supplementing PAM every third day of culture. This model of inducing redox stress was used previously (Figure 2) [28,42]. NTP generates short-lived reactive species such as OH radicals that are not stable in PAM. These short-lived OH species lead to the formation of H_2_O_2_ (long-lived species), which can initiate apoptosis and can be applied to treat tissues where direct plasma treatment is not possible [40]. In line with previous studies, exposure to PAM led to a decrease in the viability of fibroblasts. Nonmalignant fibroblasts tend to be more resilient to redox stress than cancer cells [43]. High-dose concentration (50% PAM) exposure was required to induce a 20% reduction in metabolic activity in acute stress conditions. This reduction was subsequently enhanced after periodic PAM exposure, but eventually led to non-significant differences in overall survival capacity (Figure 2). Such a trend was also observed for terminal cell death, indicating cumulative loss of cellular membrane integrity. Furthermore, the expression patterns of caspases and pro-apoptotic genes suggest the onset of apoptosis, which agrees with previous studies [28,43]. The expression patterns of apoptotic markers became inconsistent following 3 weeks of acute stress but eventually upregulated at week 12. This leads to speculations about the threshold capacity of fibroblast cells to neutralize oxidative stress.

Our study shows that soft jet plasma exposure generates high levels of RNS and moderate levels of ROS in the culture medium, as reported in several studies (Figure 3) [40,44]. Furthermore, supplementation of PAM to cells elevates the intracellular RONS levels, as observed in our study. Intracellular RONS levels do not increase initially but tend to accumulate over the study span after periodic redox stress. Several studies have reported a non-significant increase in intracellular RONS levels in nonmalignant compared to malignant cells [17,45] in acute stress conditions, while accumulation of intracellular RONS is reported in a study using the redox challenge model [28]. Cells have developed antioxidant defense systems that counteract the imbalance of reactive species and protect the cell from stress and adverse effects. This intracellular defense system is primarily composed of nonenzymatic antioxidants (glutathione) and antioxidant enzymes (glutathione peroxidase, GPx; glutathione reductase, GR; catalase; superoxide dismutase, SOD). These antioxidants work together to eliminate most free radicals. Our study shows the upregulation of antioxidant enzymes concordant with elevated intracellular RONS levels. We show increased expression levels of catalase and SOD, similar to increases in SOD1 and SOD3 reported previously [28]. This study also shows an increase in mitochondrial SOD2 (MnSOD) following periodic redox challenge. SODs rapidly convert superoxide anion radicals into more stable and less reactive H_2_O_2,_ while GPx concurrently reduces H_2_O_2_ to water and oxygen. Catalase expression remained unaffected apart from a significant but very modest increase at week 12. Although protein expression of these redox enzymes was not investigated in this study, we speculate that the cells try to recalibrate themselves for maintaining homeostasis after 6 weeks of redox challenge, resulting in an adjusted expression of antioxidant enzymes despite elevated RONS levels. This is in line with a previous study finding of altered transcriptional profiles in cells under repeated redox stress conditions over several weeks [28].

Changes in antioxidant systems coordinate and regulate the flux of energy metabolic pathways [46]. Low doses of reactive species can stimulate cellular proliferation by enhancing energy output, while high doses of reactive species can inhibit proliferation by affecting cellular and mitochondrial energetics [43,47,48]. As described previously, 25% and 50% PAM exposure did not reduce ATP levels in fibroblasts, while 100% PAM decreased the ATP levels significantly (Figure 4A and Figure S4). Moreover, ATP levels remained unaffected throughout the investigation span despite periodic PAM exposure. ATP synthesis is mainly carried out by ATP synthase enzymes encoded by the ATP5A gene. ATP5A expression initially increased at low PAM dose but remained unaffected thereafter. The initial temporal increase can be seen as an attempt to promote cellular proliferation reported in previous studies. However, this increase was not sufficient for a significant increase in viability (Figure 2). ATP production is dependent on mitochondrial integrity and function. We assessed mitochondrial structural integrity and function by evaluating mitochondrial membrane potential. In our study, mitoflow intensity was enhanced in PAM-treated groups. Such changes in mitochondrial membrane potential have been observed previously following plasma treatment [24,49,50,51]. DeltaPsi fluctuations initially altered greatly, followed by stabilization at 6 weeks of periodic PAM exposure and increased again eventually. Similar alterations in mitochondrial membrane potential due to RONS levels were observed exhibiting correlation of mitochondrial numbers and energetics after plasma treatment [50,51]. Taken together, mitochondrial SOD expression, ATP synthesis, and mitochondrial membrane potential indicate the presence of a compensatory mechanism within mitochondria of fibroblasts. Understanding this mechanism could help to draw several implications in redox-based skin diseases.

Imbalance in reactive species and the resultant oxidative stress can affect cell cycle regulation and induce apoptosis [52]. Cell cycle progression is mainly dependent on cyclins, cyclin-dependent kinases, and their inhibitors [53,54]. In addition, these proteins work closely with key tumor suppressors such as p53. We observed cell cycle arrest after 12 weeks of periodic redox stress (Figure 5). We evaluated the expression patterns of cell cycle regulators and observed a consistent increase in Cdkn1a, Cdkn2a, and p53. These proteins play important roles in G1/G0 and G2/M arrest. p21 protein encoded by cdkn1a is responsible for CDK/cyclin inhibition and cell cycle arrest mostly in the G1/G0 phase. Cdkn2a-encoded protein, however, is capable of inducing cell cycle arrest in G1 and G2 phases. Cdkn2a can bind and inhibit MDM2-mediated degradation of p53. This may explain enhanced levels of p53 expression followed by p53-dependent transactivation leading to apoptosis [55]. However, we observed cell cycle arrest mostly in the G2 phase. We speculate that cdkn1a induces apoptosis by inhibiting cyclinB1/cdc2 complexes in a p53-independent mechanism in our system. The increase in p53 expression may be significant along with the DNA damage response because of redox stress [22,56,57]. DNA damage responses progress with an increase in redox stress, as indicated by H2AX and ATM expression patterns. Although protein signaling and the downstream mechanism were not investigated, the onset of DNA damage response was evaluated and illustrated by the upregulation of H2AX and ATM. Moreover, the expression patterns of cell cycle regulators and DNA damage response markers are dysregulated after the sixth week of redox stress, consistent with other assays, indicating restabilizing mechanisms employed by fibroblasts. When redox stress levels increase beyond the threshold of fibroblasts, these markers become enhanced to induce cell cycle arrest or apoptotic cell death.

**Figure 6:**
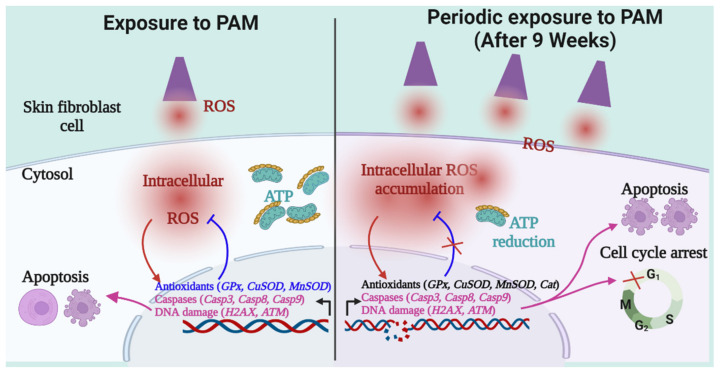
An illustration depicting the impact of periodic redox stress in skin fibroblasts. This figure is prepared using licensed BioRender online tool.

Collectively, this study demonstrates the physiological changes altered as a result of periodic stress in fibroblasts, as illustrated in Figure 6. Fibroblasts are able to adapt well against periodic oxidative stress until 9 weeks through inherent mechanisms, an area for future investigations. Periodic oxidative stress after 9 weeks tends to surpass the ability of fibroblasts to maintain their homeostatic physiological conditions, leading to the accumulation of excessive DNA damage, cell cycle arrest, and dysregulated transcriptome. The period and frequency of nonthermal plasma are important factors to consider before its translation into clinical use.

## 4. Materials and Methods

### 4.1. Cell Culture

A human dermal fibroblast cell line, GM00637 (GM), was purchased (Coriell Institute for Medical Research, Camden, NJ, USA). Cells were cultured in MEM (LM007-07; Welgene, Gyeongsan, Korea) with 10% fetal bovine serum (A500-500, RDtech, Parlin, NJ, USA) without inactivation, and an antibiotic combination of streptomycin (100 μg/mL) and penicillin (100 U/mL) (LS203-01; Welgene, Gyeongsan, Korea). Cells were maintained in an incubator with 5% CO_2_ at 37 °C and appropriate humidity. Cells were sub-cultured every two to three days until the commencement of the experiment. Cells were tested for mycoplasma contamination before the start of the experiment and in the middle stage (week 6) of the scheduled experiment.

### 4.2. PAM Generation and Treatment

To begin the experiment, GM fibroblast cells were seeded at 1 × 10^6^ cells in a T-75 cell culture flask and allowed to grow in a complete medium for 2 days. On the third day, 3 mL/well of complete MEM medium was exposed to soft jet plasma for 200 s. PAM was used to replace the complete medium in the T-75 cell flask. Three PAM groups (in duplicates) were designed beside the control with no medium replacement: 25%, containing 25% plasma-activated medium and 75% complete medium; 50%, containing equal proportions of plasma-activated medium and complete culture medium; and 100% PAM, used to replace the culture medium without dilution. On the 4th day, cells were sub-cultured at 1 × 10^6^ cells in a T-75 cell culture flask and allowed to grow in a complete medium, marking the end of one plasma treatment cycle. This cyclic process was repeated, and samples were collected on the 4th day of weeks 0, 3, 6, 9, and 12 from the day of the first PAM replacement.

### 4.3. Cell Growth Assay

On the 4th day of the plasma treatment cycle at weeks 0, 3, 6, 9, and 12, GM cells were seeded at a density of 1 × 10^4^ in 96-well plates with a standard complete medium. After 24 h, the viability of cells was determined using the alamarBlue dye (DAL1025; Thermo Fisher Scientific, Waltham, MA, USA). Viability was calculated by the measurement of fluorescence indicative of alamarBlue conversion, as explained previously [50,58].

### 4.4. Terminal Cell Death

Changes in the cell death rate after culturing cells with PAM over time were determined by measuring the PI (P4170; Sigma Aldrich, St. Louis, MO, USA) uptake. PI staining solution (50 ng/mL) was mixed in PBS buffer (LB001-02_500mL; Welgene, Gyeongsan, Korea). For this experiment, 2 × 10^5^ cells from each group were seeded on the 4th day of the plasma treatment cycle in 35 mm dishes in triplicates. After 24 h, the cells were subjected to PBS washing, trypsinization using 0.25% trypsin-EDTA (SH30042.01; HyClone, Logan, UT, USA), and harvesting. The harvested cell sample was stained using PI staining solution. Data acquisition and analysis were carried out using FACS Verse (BD Biosciences, Franklin Lakes, NJ, USA).

### 4.5. Quantitative Real-Time PCR Analysis

For this assay, RNA from cells grown in the control flask and cells cultured in PAM were extracted using Trizol reagents (RNAiso Plus (9109), Takara, Japan) following the protocol provided by the manufacturer. Total RNA (2 µg) was used to synthesize the cDNA template using MMLV Reverse Transcriptase supermix kit (RT001M, Enzynomics, Daejeon, Korea) components on a thermocycler (Applied Biosystems, Waltham, MA, USA), as per the manufacturer’s protocol. For quantitative qPCR, reactions were set up using iQ SYBR Green Supermix (170-8882; Bio-Rad, Hercules, CA, USA) on a real-time detection (iCycler IQ) system (Bio-Rad, Hercules, CA, USA). All primers listed in Table 1 were designed and purchased from DNA Macrogen (Seoul, Korea).

### 4.6. Reactive Species (RONS) Detection

To estimate the concentration of reactive species in the medium after NTP exposure, 3 mL per well of complete MEM medium was exposed to soft jet plasma for 200 s in 12-well plates. Then, 100 uL was taken for each PAM group, and samples were processed promptly for H_2_O_2_ and NO_x_ detection following the manufacturer protocol for the QuantiChrom™ Peroxide Assay Kit (DIOX-250, BioAssay systems, Hayward, CA, USA) and the QuantiChrom Nitric Oxide Assay Kit (D2NO-100; BioAssay systems, Hayward, CA, USA), respectively. Absorbance was measured using the Synergy HT (BioTek Instruments) plate reader. Untreated complete medium was used as the control for estimation. To detect the intracellular RONS, 2 × 10^5^ cells from each group were seeded on the 4th day of the plasma treatment cycle of designated week time points in 35 mm dishes in triplicates. After 24 h, cells from all PAM groups were harvested and incubated with H_2_-DCF-DA (D-399; Invitrogen, Waltham, MA, USA) reagent for 30 min. After incubation, cells were washed with 1X cold PBS, acquired immediately using a BD FACS Verse flow cytometer, and analyzed by FACS suite software.

### 4.7. Cell Cycle Analysis

Next, 2 × 10^5^ cells/well were seeded on the 4th day of the plasma treatment cycle at designated time points in 35 mm dishes. After 24 h of incubation, cells were harvested, washed with cold PBS supplemented with 2% fetal bovine serum, fixed with ethanol (70%) for 1 h, and centrifuged. The pellet was resuspended in PI staining solution (20 µg/mL PI and 50 µg/mL RNase A) and incubated overnight at 4 °C. The following day, cells were centrifuged, washed, and then resuspended in cold 1X PBS for sample acquisition with BD FACS Verse and analysis with FACS suite software.

### 4.8. Cell Energetics Assay (ATP Measurement)

Cellular ATP indicates cellular health levels. It was estimated following the manufacturer’s protocol of Promega Cell Titer-Glo Assay (G7572, Promega, Madison, WI, USA). To this end, we seeded 5 × 10^3^ cells per well of 96-well plates with the volume of 100 µL on the 4th day at sampling week time point. After 24 h of incubation, an equal volume of prewarmed reagent was added to cells, followed by incubation for 1 h at 37 °C. After 1 h incubation, we measured luminescence using a microplate reader (BioTek, Santa Clara, CA, USA).

### 4.9. Flow Cytometry for Mitochondria Membrane Potential

Mitoflow reagent (4004; Cell technology, Davis, CA, USA) was used to analyze Δψm using a flow cytometric analysis assay, as previously described [39]. Briefly, cells from the control flask and PAM cultured cells were harvested and stained with mitoflow reagent following the manufacturer’s protocol. Samples were immediately analyzed using a BD FACSVerse system equipped with the FACS suite software.

### 4.10. Confocal Microscopy

Microscopic analysis for DNA damage was performed using DAPI staining. Briefly, 2 × 10^5^ cells per cover slide were seeded in 35 mm dishes on the 4th day of each week time point. After 24 h of incubation, cells were processed for washing with cold PBS, then fixated with 2% paraformaldehyde (P2031; Biosesang, Seongnam, Korea) for 30 min at room temperature, followed by washing again with cold PBS, and eventually stained with DAPI for 10 min. Then, nuclear morphology was assessed by confocal microscopy.

### 4.11. Statistics

Experimental data values were expressed as the mean ± SD of three biological replicates. Statistical comparison was performed using either two-tailed unpaired parametric *t*-tests among two groups or one/two-way ANOVA with Dunnett or Tukey corrections for multiple comparisons to the untreated control, as per the experimental conditions using PRISM9 software. Differences were indicated as statistically significant if * *p* < 0.05, ** *p* < 0.01, or *** *p* < 0.001. We have mentioned the statistical methods performed in respective figure legends for clarity.

## 5. Conclusions

In this study, we investigated the extent of changes in skin fibroblast cells under periodic redox stress conditions in vitro. We observed alterations in cellular processes involved in cellular maintenance and redox stress tolerance. Based on the obtained results, we recommend that any investigations using periodic stress by NTP with natural air be limited to less than 9 weeks in the fibroblast cell system to avoid cell damage. We believe these results could help narrow the knowledge gap and highlight the need to understand fibroblast plasticity that may help implement NTP medicine in redox-related skin diseases. Further studies using animal models focusing on these mechanisms are required to better understand and develop NTP therapy in clinical use.

## Figures and Tables

**Table 1:** List of primers used in this study.

Gene Name	Sequence (5′-3′)
ACTIN-forward	GGC ATC CTC ACC CTG AAG TA
ACTIN-reverse	AGG TGT GGT GCC AGA TTT TC
Casp3-forward	ATG TCG ATG CAG CAA ACC TC
Casp3-reverse	TCC TTC TTC ACC ATG GCT CA
Casp8-forward	CCC AAA TCA ACA AGA GCC TGC
Casp8-reverse	TCA GAC AGT ATC CCC GAG GTT
BCL-XL-forward	TCA GTG AGT GAG CAG GTG TT
BCL-XL-reverse	GGC CTC AGT CCT GTT CTC TT
BAX-forward	AAG AAG CTG AGC GAG TGT CTC
BAX-reverse	GCT GGC AAA GTA GAA AAG GGC
GPx-forward	TTG ACA TCG AGC CTG ACA TC
GPx-reverse	CAA GGT GTT CTT CCC TCG TA
CAT-forward	TCT GGA GAA GTG CGG AGA TT
CAT-reverse	AGT CAG GGT GGA CCT CAG TG
CuZnSOD-forward	GAA GGT GTG GGG AAG CAT TA
CuZnSOD-reverse	ACA TTG CCC AAG TCT CCA AC
MnSOD-forward	TGT ACC GGT TCC GAG TTT TC
MnSOD-reverse	TTC AGG CCC TAC AAT TCA CC
ATP5A-forward	TTT TGC CCA GTT CGG TTC TG
ATP5A-reverse	GAT ATC CCC TTA CAC CCG CA
p53-forward	GCC CCT CCT CAG CAT CTT ATC
p53-reverse	AAA GCT GTT CCG TCC CAG TAG
H2AX-forward	CAA CAA GAA GAC GCG AAT CA
H2AX-reverse	CGG GCC CTC TTA GTA CTC CT
ATM-forward	TCC GTC AGC AAA GAA GTA GAA
ATM-reverse	TGG GAT AGA GCG AAT ACA CAG
Cdkn1A-forward	TTG GCT CCC CTG TAC CTT TT
Cdkn1A-reverse	CCT TCC CCT TCC AGT CCA TT
Cdkn2A -forward	CCC AAC GCA CCG AAT AGT TA
Cdkn2A -reverse	ACC CCT TCT GAA AAC TCC CC
Casp9-forward	CGA CAT CTT TGA GCA GTG GG
Casp9-reverse	GAA AGC TTT GGG GTG CAA GA

## Supplementary Materials

The following supporting information can be downloaded at: https://www.mdpi.com/article/10.3390/ijms23063120/s1.
