# Supplementary material for: Periodic Exposure of Plasma-Activated Medium Alters Fibroblast Cellular Homoeostasis
Source: Int J Mol Sci. 2022 Mar 14;23(6):3120. doi: 10.3390/ijms23063120 (PMC8949019; doi:10.3390/ijms23063120)
Supplement: Supplementary file 1 [file ijms-23-03120-s001.zip › ijms-1597218-supplementary.pdf]

International Journal of
Molecular Sciences

MDPI

Supplementary Information

Periodic Exposure of Nonthermal Plasma Activated Medium Impairs Fibroblast Cellular Homoeostasis

Pradeep Bhartiya [1], Neha Kaushik [2], Linh N. Nguyen [1], Sander Bekeschus [3], Kai Masur [3], Klaus-Dieter Weltmann [3], Nagendra K. Kaushik [1,*], Eun H. Choi [1,*]

1 Department of Electrical and Biological Physics, Plasma Bioscience Research Center, Kwangwoon University, Seoul 01897, Korea; pradeepindian65@gmail.com (P.B.); nhatlinhusth@gmail.com (L.N.N.)
2 Department of Biotechnology, College of Engineering, The University of Suwon, Hwaseong 18323, Korea; neha.bioplasma@gmail.com
3 Laboratory of Plasma Technology, Institute of Materials Science, Vietnam Academy of Science and Technology, 18 Hoang Quoc Viet, Hanoi 100000, Vietnam
4 ZIK Plasmatis, Leibniz Institute for Plasma Science and Technology, 17489 Greifswald, Germany; sander.bekeschus@inp-greifswald.de (S.B.); kai.masur@inp-greifswald.de (K.M.); weltmann@inp-greifswald.de (K.-D.W.)
* Correspondence: kaushik.nagendra@kw.ac.kr (N.K.K.); ehchoi@kw.ac.kr (E.H.C.)

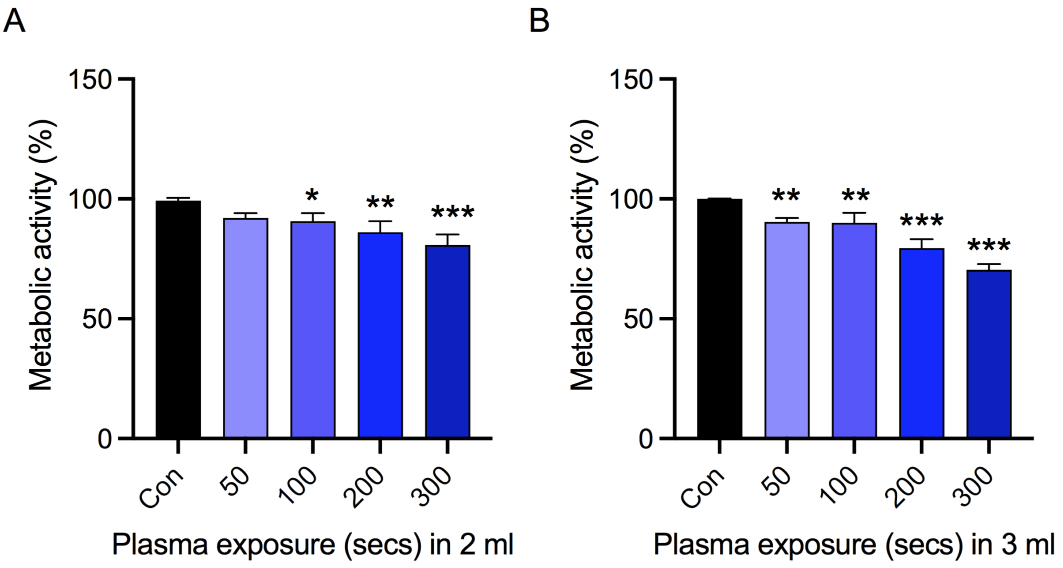

Figure S1. Standardization of plasma exposure time. Measurement of metabolic activity (percentage) in GM cells after exposure to nonthermal plasma for indicated exposure times in (A) 2 ml and (B) 3 ml of complete MEM medium. Statistical significance was evaluated using one-way ANOVA using Dunnett's multiple comparision test and is indicated as *$p < 0.05$, **$p < 0.01$ and ***$p < 0.001$.

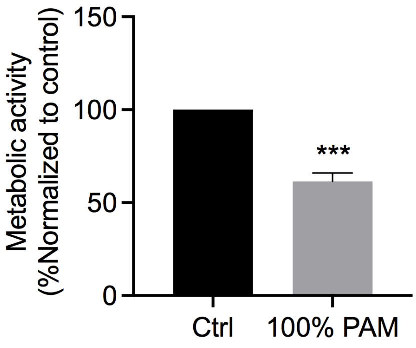

Figure S2. Evaluation of cytotoxicity in fibroblast cells after PAM treatment. Representative graph showing metabolic activity levels in GM cells after PAM treatment. Statistical significance was evaluated using Two-tailed unpaired parametric t-test and denoted as ****p* < 0.001.

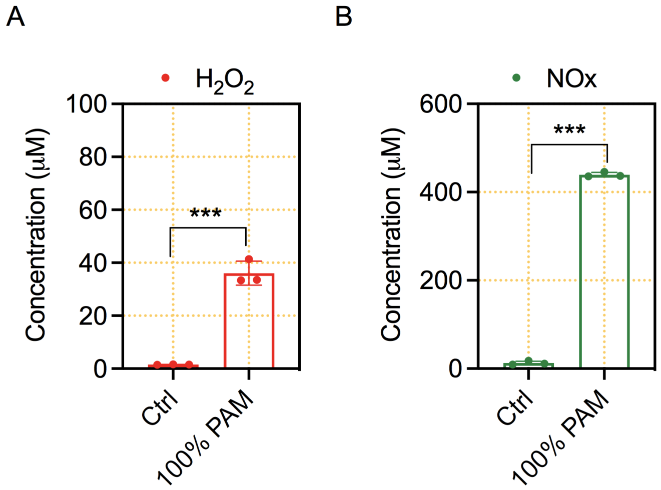

Figure S3. Measurement of reactive species in PAM which was used to treat fibroblast cells. Representative graphs indicating levels of (**A**) H_2O_2 and (**B**) NO_x in PAM. Significance was evaluated using Two-tailed unpaired parametric t-test and indicated as ****p* < 0.001.

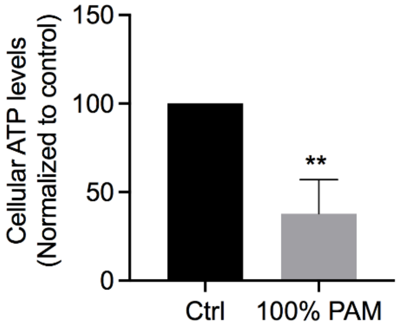

Figure S4. Evaluation of ATP levels in fibroblast cells. Representative graph indicating changes in ATP levels in control and PAM treated GM cells. Statistical significance was evaluated using Two-tailed unpaired parametric t-test and is denoted as ***p* < 0.01.

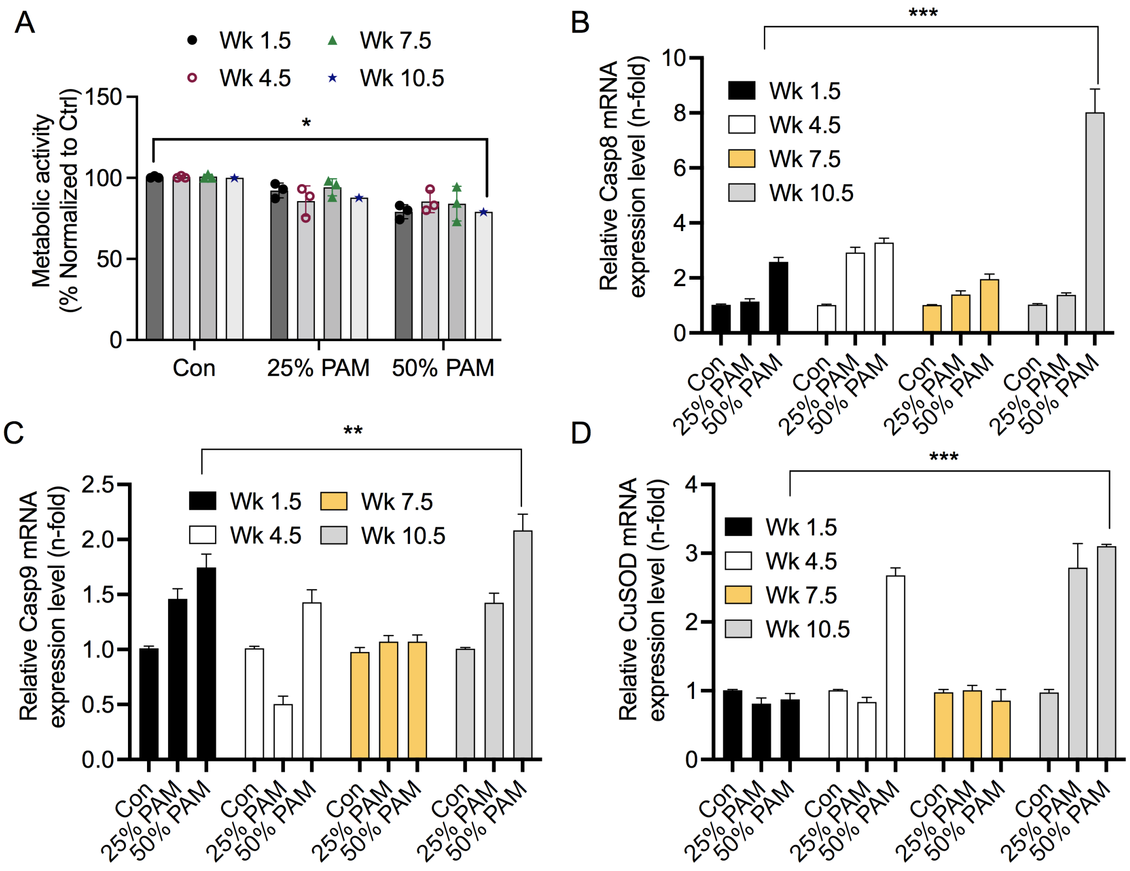

Figure S5. Effect of periodic exposure at intermediate weeks on fibroblast cells. (**A**) Bar graph representing metabolic activity of fibroblasts after periodic PAM exposure for intermediate weeks (Wks). Evaluation of cell death markers (**B**) Casp8 and (**C**) Casp9 in fibroblasts after periodic stress at intermediate Wk. (**D**) Graph indicating mRNA expression levels of CuSOD after periodic exposure for intermediate week timepoints. Statistical significance was evaluated using two-way ANOVA using Tukey's multiple comparision test and is indicated as *$p < 0.05$, **$p < 0.01$, and ***$p < 0.001$.
